# Optimal spirometry thresholds for the prediction of chronic airflow obstruction: a multinational longitudinal study

**DOI:** 10.1183/23120541.00624-2024

**Published:** 2025-03-03

**Authors:** Abby H.S. Lam, Sheikhah A. Alhajri, James Potts, Imed Harrabi, Mahesh Padukudru Anand, Christer Janson, Rune Nielsen, Dhiraj Agarwal, Andrei Malinovschi, Sanjay Juvekar, Meriam Denguezli, Thorarinn Gislason, Rain Jõgi, Vanessa Garcia-Larsen, Rana Ahmed, Asaad Ahmed Nafees, Parvaiz A. Koul, Althea Aquat-Stewart, Peter Burney, Ben Knox-Brown, Andre F.S. Amaral

**Affiliations:** 1 National Heart and Lung Institute, Imperial College London, London, UK; 2 Ibn El Jazzar Faculty of Medicine of Sousse, University of Sousse, Sousse, Tunisia; 3 Department of Respiratory Medicine, JSS Medical College, JSSAHER, Mysuru, India; 4 Department of Medical Sciences: Respiratory, Allergy and Sleep Research, Uppsala University, Uppsala, Sweden; 5 Department of Thoracic Medicine, Haukeland University Hospital, Bergen, Norway; 6 Department of Clinical Science, University of Bergen, Bergen, Norway; 7 Vadu Rural Health Program, KEM Hospital Research Centre, Pune, India; 8 Dr. D. Y. Patil Medical College, Hospital and Research Centre, Dr. D. Y. Patil Vidyapeeth, Pune, Maharashtra, India; 9 Laboratoire de Recherche en Physiologie de l'Exercice et Physiopathologie, de l'Intégré au Moléculaire (LR19ES09), Faculté de Médecine de Sousse, Université de Sousse, Sousse, Tunisia; 10 Faculty of Medicine, University of Iceland, Reykjavik, Iceland; 11 Department of Sleep, Landspitali - The National University Hospital of Iceland, Reykjavik, Iceland; 12 Tartu University Hospital, Lung Clinic; 13 Department of International Health, John Hopkins Bloomberg School of Public Health, Baltimore, MD, USA; 14 The Epidemiological Laboratory, Khartoum, Sudan; 15 Department of Community Health Sciences, Aga Khan University, Karachi, Pakistan; 16 Department of Pulmonary Medicine, Sheri Kashmir Institute of Medical Sciences, Srinagar, Jammu and Kashmir, India; 17 Department of Medicine, University of the West Indies, Kingston, Jamaica; 18 Cambridge Respiratory Physiology, Royal Papworth & Cambridge University Hospitals NHS FT, Cambridge, UK; 19 NIHR Imperial Biomedical Research Centre, London; 20 Royal Commission Hospital in Jubail, Jubail, Saudi Arabia; 21 Joint first authors; 22 Joint senior authors

## Abstract

**Introduction:**

Chronic airflow obstruction is key for COPD diagnosis, but strategies for its early detection are limited. We aimed to define the optimal z-score thresholds for spirometry parameters to discriminate chronic airflow obstruction incidence.

**Methods:**

The Burden of Obstructive Lung Disease study is a multinational cohort study. Information on respiratory symptoms was collected and pre- and post-bronchodilator spirometry was performed at baseline. 18 study sites were followed-up with repeat measurements after a median of 8.4 years. We converted lung function measurements into z-scores using the Third National Health and Nutrition Survey reference equations. We used the Youden index to calculate the optimal z-score thresholds for discriminating chronic airflow obstruction incidence. We further examined differences by smoking status.

**Results:**

We analysed data from 3057 adults (57% female, mean age: 51 years at baseline). Spirometry parameters were good at discriminating chronic airflow obstruction incidence (area under the curve 0.80–0.84), while respiratory symptoms performed poorly. The optimal z-score threshold was identified for pre-bronchodilator forced expiratory volume in 1 s to forced vital capacity ratio (FEV_1_/FVC) <−1.336, equivalent to the 9th percentile (sensitivity: 78%, specificity: 72%). All z-score thresholds associated with a lower post-bronchodilator FEV_1_/FVC and greater odds of chronic airflow obstruction at follow-up. The risk of chronic airflow obstruction was slightly greater for current smokers and, to some extent, never-smokers with a pre-bronchodilator FEV_1_/FVC <9th/10th percentiles at baseline, particularly among males.

**Conclusions:**

Spirometry is better than respiratory symptoms at predicting chronic airflow obstruction incidence. A pre-bronchodilator FEV_1_/FVC <9th/10th percentiles, particularly among current smokers, could suggest early airflow obstruction or pre-COPD.

## Shareable abstract

Spirometry is better than respiratory symptoms at predicting chronic airflow obstruction incidence. A pre-bronchodilator FEV_1_/FVC <9th/10th percentiles, particularly among current smokers, could suggest early airflow obstruction or pre-COPD

## Introduction

Globally, COPD is a leading cause of morbidity and mortality [1, 2]. In addition to respiratory symptoms, a primary feature of COPD is chronic airflow obstruction, which is defined by an abnormal post-bronchodilator forced expiratory volume in 1 s to forced vital capacity ratio (FEV_1_/FVC) [3]. Strategies for the early detection of COPD are limited [4], despite its pathogenesis starting years before airflow obstruction is detectable [5].

There has been increasing interest in the predictive ability of spirometry parameters and respiratory symptoms to identify those at risk of COPD [6–9], a physiological state often referred to as pre-COPD [10]. Recently, Tan *et al.* [6], using data from the Tasmanian Longitudinal Health Study, identified optimal z-score thresholds for spirometry parameters to predict incidence of chronic airflow obstruction. They found that spirometry was a better predictor than respiratory symptoms and, furthermore, that pre-bronchodilator FEV_1_/FVC below the 10th percentile performed best. In addition, it has been shown that having a FEV_1_/FVC <0.75 is associated with incidence of chronic airflow obstruction in the Lovelace smokers cohort [9], as has having a mean forced expiratory flow rate between 25% and 75% of the FVC (FEF_25–75_) below the 20th percentile in a patient population in South Korea [7]. These data suggest that relying solely on the lower limit of normal (LLN), which is equivalent to the 5th percentile of a normal non-smoking population, may fail to identify all those with disease.

Before the thresholds identified by Tan *et al.* [6] can be implemented in clinical practice, it is important to externally validate their findings. Using longitudinal data from the multinational Burden of Obstructive Lung Disease (BOLD) study, we aimed to identify the optimal spirometry z-score thresholds for FEV_1_/FVC and FEF_25–75_, for predicting incidence of chronic airflow obstruction. In addition, we aimed to compare these to the discriminative ability of respiratory symptoms alone, and further, to the thresholds identified by Tan *et al.* [6] and Kwon *et al.* [7].

## Materials and methods

### Study subjects

BOLD is a multinational observational cohort study. The protocol for both phases of data collection have been published previously [11, 12]. At baseline, non-institutionalised adults ≥40 years of age were recruited from 41 municipalities across 34 countries, between January 2003 and December 2016. Site-specific sampling strategies were implemented to recruit representative samples of the populations studied. Participants from 18 sites were followed up between January 2019 and October 2021. For the present study, we used longitudinal data from BOLD, participants were included if they had completed the study core questionnaire, had acceptable and repeatable pre- and post-bronchodilator spirometry, and no evidence of chronic airflow obstruction at baseline, and had acceptable post-bronchodilator spirometry at follow-up. Ethical approval was obtained by each site from the local ethics committee, and informed consent was obtained from every participant. All sites followed good clinical practice and local ethics regulations.

### Data collection

Demographic data and information on respiratory symptoms, health status and exposures were collected using standardised questionnaires translated into the local language. For the present study, dyspnoea was assessed using the Modified Medical Research Council dyspnoea scale, where participants rated their breathlessness according to five grades: Grade 0—dyspnoea only with strenuous exercise; Grade 1 – dyspnoea when hurrying on the level or up a slight hill; Grade 2 – dyspnoea when walking at own pace on the level; Grade 3 – dyspnoea when walking 100 yards or for a few minutes; Grade 4 – too short of breath to leave the house or short of breath when dressing or undressing. We generated a binary variable where a grade of 0–1 indicates no/minimal breathlessness, and a grade ≥2 indicates significant breathlessness. Presence of chronic cough, chronic phlegm and wheeze was determined by positive responses to the following questions: 1) “do you cough on most days for as much as 3 months each year?”; 2) “do you bring up phlegm on most days for as much 3 months each year?”; and 3) “have you had wheezing or whistling in the chest at any time in the last 12 months?” Lung function, including FEV_1_, FVC and FEF_25–75_, was measured using the ndd EasyOne Spirometer (ndd Medizintechnik AG, Zurich, Switzerland), before and 15 min after 200 µg inhaled salbutamol. Spirograms were centrally reviewed and assigned a quality score based on acceptability and repeatability criteria [13]. Pre- and post-bronchodilator measurements for FEV_1_/FVC and FEF_25–75_ were converted to z-scores in the RStudio *Rspiro* package using reference values for European Americans in the Third US National Health and Nutrition Examination Survey (NHANES) [14]. The use of European American reference equations is in line with previous BOLD publications [3, 15–17]. NHANES equations have also been shown to give similar prevalence estimates for airflow obstruction regardless of race correction [18], while recent evidence suggests that race correction may misclassify individuals with underlying disease [19].

### Outcome measure

The outcome of interest was the presence of chronic airflow obstruction at follow-up defined as a post-bronchodilator (15 min after 200 µg inhaled salbutamol) FEV_1_/FVC less than the LLN according to reference equations for European Americans from the NHANES study [14].

### Statistical analysis

From baseline spirometry, optimal z-score thresholds for the discrimination of chronic airflow obstruction at follow-up were identified using the unweighted Youden index [20]. Sensitivity, specificity, likelihood ratios and Youden index at the optimal threshold (Ymax) were reported for each spirometry parameter. We repeated these analyses investigating the accuracy of baseline respiratory symptoms, including dyspnoea, chronic cough, chronic phlegm and wheeze, to discriminate incidence of chronic airflow obstruction at follow-up. To investigate whether the thresholds changed when using different reference equations, we repeated our analysis using reference equations for Caucasians from the Global Lung Initiative (GLI) [21].

To estimate the association between being below the optimal spirometry thresholds and incidence of chronic airflow obstruction at follow-up, we performed multilevel logistic regression analyses. We fitted the models with a random intercept to account for clustering by study site and a random slope to allow the association of the optimal thresholds with incident chronic airflow obstruction to vary across sites. We also used multilevel linear regression to investigate whether being below the optimal thresholds was associated with a lower post-bronchodilator FEV_1_/FVC. We adjusted for sex (male/female), age (years), body mass index (kg·m^−2^), smoking status (never/former/current) and pack-years of smoking. For the FEF_25–75_ thresholds, we also adjusted for variation in the FVC [22]. We did not adjust for follow-up time as this was determined at site level. We additionally investigated whether there was an interaction between smoking status and being below the optimal thresholds on incidence of chronic airflow obstruction.

We constructed receiver operating characteristic curves and calculated the area under the curve (AUC) to compare the discriminative ability of the optimal spirometry thresholds identified by this study to those of Tan *et al.* [6] and Kwon *et al.* [7]. To assess the overall predictive performance of the parameters, we calculated the Brier score, which ranges between 0 and 1, with 0 indicating a perfect prediction and 1 a non-informative predictive model [23]. We performed sensitivity analyses investigating the association between different percentile thresholds and incidence of chronic airflow obstruction, and also whether adding a history of at least one respiratory symptom at baseline to the models improved discrimination. Analyses were conducted using inverse probability weights to account for missing data at follow-up [24]. All results were considered significant if the p-value was below 0.05. Analyses were performed using Stata 17 (Stata Corp., College Station, TX, USA).

## Results

**FIGURE 1:**
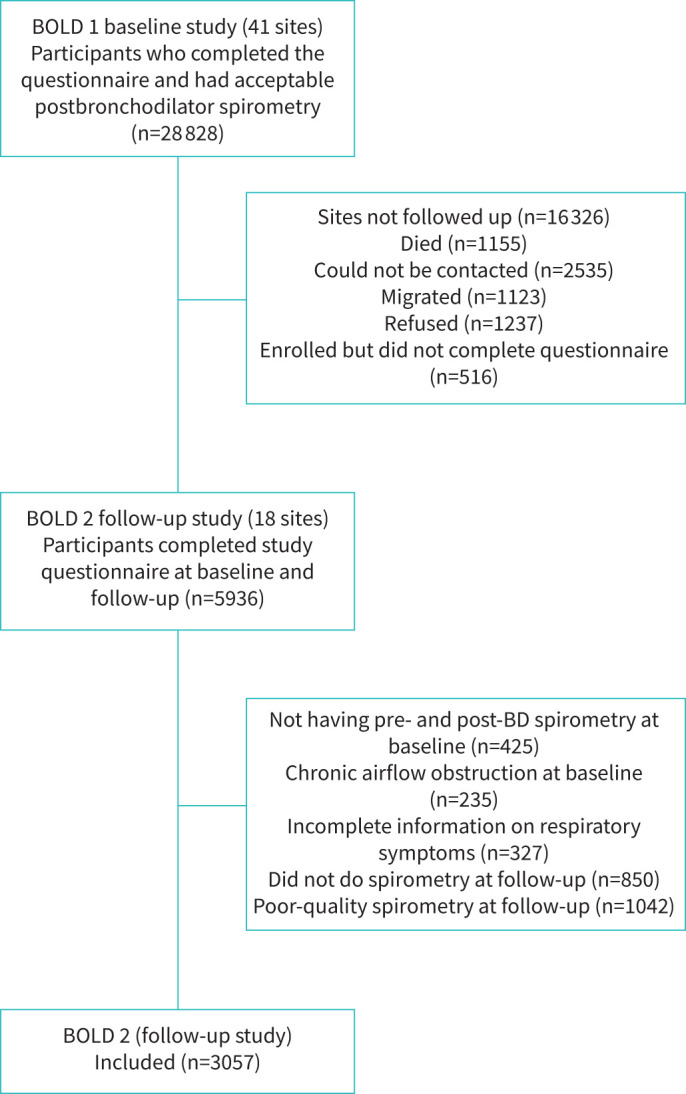
Study flow diagram. BD: bronchodilator.

18 study sites collected data at baseline and follow-up with 12 502 eligible participants. At follow-up, 1155 participants had died, 3658 had migrated or were unreachable, 1237 refused to participate and 516 enrolled but never completed the core questionnaire. 5936 participants completed the core questionnaire at baseline and follow-up. From the baseline data, 987 participants were excluded due to not having both pre- and post-bronchodilator spirometry measurements (n=425), having chronic airflow obstruction (n=235) or having incomplete information on respiratory symptoms (n=327). From the follow-up data, 1892 participants were excluded due to not performing spirometry (n=850) or having poor-quality spirometry (n=1042) (figure 1). A total of 3057 participants with a median (IQR) follow-up time of 8.4 years (6.2–11.0) were included in our analyses.

The characteristics of study participants are displayed in table 1. Females made up 57% of the study population (1736 of 3057). Mean age at baseline ranged from 46 years in Mysore, India, to 61 years in Tartu, Estonia. The prevalence of never-smoking at baseline ranged from 34% (57 of 169) in Bergen, Norway, to 99% (73 of 74) in Sémé-Kpodji, Benin. Reporting at least one respiratory symptom at baseline was least common in Kashmir, India (0%, 0 of 15) and most common in Sousse, Tunisia (60%, 57 of 95). Median follow-up time ranged from 4.4 years (IQR: 4.0–4.7) in Karachi, Pakistan, to 15.5 years (IQR: 14.6–15.8) in Bergen, Norway. Over the follow-up period, 131 of 3057 participants (4%) developed chronic airflow obstruction. Incident chronic airflow obstruction was lowest in Fes, Morocco (0%, 0 of 15) and Sémé-Kpodji, Benin (0%, 0 of 74), and highest in Tartu, Estonia (9%, 11 of 121).

**TABLE 1:** Baseline characteristics of study participants (n=3057)

Country (city)	n	Female, n (%)	Age years, mean±sd	Never- smoker, n (%)	Pre-BD FEV_1_/FVC z-score, mean±sd	Post-BD FEV_1_/FVC z-score, mean±sd	Pre-BD FEF_25–75_ z-score, mean±sd	Post-BD FEF_25–75_ z-score, mean±sd	Dyspnoea, n (%)	Chronic cough, n (%)	Chronic phlegm, n (%)	Wheeze, n (%)	At least one respiratory symptom, n (%)	Follow-up time years, median (IQR)	CAO at follow-up, n (%)
**Benin (Sémé-Kpodji)**	74	41 (55)	50.6±8.0	73 (99)	−0.26±0.82	0.23±0.65	−1.13±0.79	−0.65±0.72	15 (20)	0 (0)	1 (1)	2 (3)	16 (22)	7.0 (6.9–7.1)	0 (0)
**Estonia (Tartu)**	121	64 (53)	60.8±9.8	72 (60)	−0.19±1.00	0.32±0.83	−0.19±0.93	0.36±0.92	17 (14)	8 (7)	12 (10)	31 (26)	50 (41)	10.7 (10.2–11.3)	11 (9)
**Iceland (Reykjavik)**	190	93 (49)	50.9±7.7	79 (42)	0.51±0.84	0.10±0.76	−0.47±0.96	0.09±1.03	13 (7)	10 (5)	9 (5)	42 (22)	62 (33)	14.6 (14.3–13.8)	12 (6)
**India (Kashmir)**	15	5 (33)	50.9±9.4	4 (27)	−0.19±0.92	0.21±0.84	−0.47±0.80	0.03±0.89	0 (0)	0 (0)	0 (0)	0 (0)	0 (0)	8.5 (8.4–8.5)	1 (7)
**India (Mysore)**	342	207 (61)	45.6±6.2	319 (93)	0.03±0.79	0.17±0.75	−1.02±1.02	−0.90±1.01	5 (1)	2 (1)	3 (1)	1 (0)	8 (2)	7.2 (6.7–7.9)	12 (4)
**India (Pune)**	415	174 (42)	50.3±8.4	378 (91)	−0.06±0.94	0.39±0.86	−0.93±1.02	−0.46±1.05	15 (4)	6 (1)	3 (1)	14 (3)	26 (6)	10.9 (10.7–11.1)	11 (3)
**Jamaica**	20	9 (45)	50.1±6.7	11 (55)	0.35±0.75	0.74±0.75	0.02±1.50	0.56±1.57	3 (15)	1 (5)	1 (5)	4 (20)	6 (30)	5.5 (5.3–5.6)	1 (5)
**Kyrgyzstan (Chui)**	252	183 (72)	51.0±7.5	189 (75)	−0.38±0.79	0.03±0.74	−0.57±0.98	−0.09±1.01	49 (19)	15 (6)	6 (2)	29 (12)	74 (29)	6.1 (6.1–6.2)	8 (3)
**Kyrgyzstan (Naryn)**	270	175 (64)	50.5±7.5	212 (79)	−0.37±0.84	0.07±0.75	−0.48±1.05	0.01±1.03	66 (24)	21 (8)	16 (9)	33 (12)	94 (35)	6.1 (6.1–6.2)	8 (3)
**Malawi (Chikwawa)**	212	114 (54)	52.5±9.5	157 (74)	−0.25±0.97	0.15±0.83	−0.64±1.04	−0.27±1.02	28 (13)	1 (1)	0 (0)	5 (2)	34 (16)	4.8 (4.4–5.0)	16 (8)
**Morocco (Fes)**	15	5 (33)	50.2±5.4	8 (53)	−0.37±0.81	0.20±0.64	−0.37±0.81	0.05±0.89	4 (27)	2 (13)	3 (20)	3 (20)	6 (40)	10.6 (10.2–10.8)	0 (0)
**Nigeria (Ife)**	311	223 (72)	54.5±11.4	284 (91.3)	−0.22±1.03	0.28±0.92	−0.97±0.98	−0.50±0.99	25 (8)	0 (0)	0 (0)	5 (2)	29 (9)	8.3 (8.2–8.4)	15 (5)
**Norway (Bergen)**	169	87 (52)	53.6±8.3	57 (34)	−0.58±0.92	−0.12±0.90	−0.52±0.93	−0.14±−0.96	10 (6)	10 (6)	9 (5)	31 (18)	47 (28)	15.5 (14.6–15.8)	9 (5)
**Pakistan (Karachi)**	155	92 (60)	49.3±12.5	118 (76)	0.12±1.00	0.41±0.93	−0.95±1.04	−0.60±1.06	76 (49)	11 (7)	12 (8)	12 (8)	82 (53)	4.4 (4.0–4.7)	2 (1)
**Philippines (Nampicuan−Talugtug)**	230	125 (54)	51.0±8.2	126 (55)	−0.11±1.07	0.39±0.88	−0.93±1.00	−0.46±1.01	35 (15)	11 (5)	14 (6)	42 (18)	73 (32)	10.7 (10.5–11.0)	14 (6)
**Sudan (Khartoum)**	18	8 (45)	48.6±8.3	12 (67)	0.00±1.00	0.24±0.69	−0.77±1.06	−0.58±0.90	4 (22)	0 (0)	0 (0)	1 (6)	4 (22)	7.4 (7.4–7.5)	1 (6)
**Sweden (Uppsala)**	153	74 (48)	54.6±8.0	61 (40)	−0.52±0.82	0.01±0.76	−0.51±0.89	−0.03±0.93	11 (7)	8 (5)	8 (5)	30 (20)	45 (29)	13.3 (13.0–13.8)	5 (3)
**Tunisia (Sousse)**	95	57 (60)	51.3±8.7	63 (66)	0.09±0.85	0.43±0.85	−0.26±1.09	0.15±1.18	42 (44)	8 (9)	16 (17)	24 (26)	57 (60)	10.6 (10.4–10.8)	5 (5)
**Overall**	3057	1736 (57)	51.3±9.0	2223 (73)	−0.21±0.94	0.21±0.83	−0.73±0.03	−0.30±1.08	418 (14)	114 (4)	113 (4)	309 (10)	713 (23)	8.4 (6.2–11.0)	131 (4)

Dyspnoea measured according to mMRC dyspnoea scale: 0–1=minimal/no breathlessness; ≥2= significant breathlessness. Chronic cough: cough on most days for 3 months each year. Chronic phlegm: phlegm on most days 3 months each year. Wheeze: wheezing or whistling in the chest at any time in the last 12 months. z-scores calculated using the Rspiro package in R based on reference equations for European Americans in The Third National Health and Nutrition Survey (NHANES III) [14]. BD: bronchodilator (200 µg salbutamol); FEV_1_/FVC: forced expiratory volume in 1 s as a ratio of the forced vital capacity; FEF_25–75_: mean forced expiratory flow rate between 25% and 75% of the forced vital capacity; CAO: chronic airflow obstruction.

**FIGURE 2:**
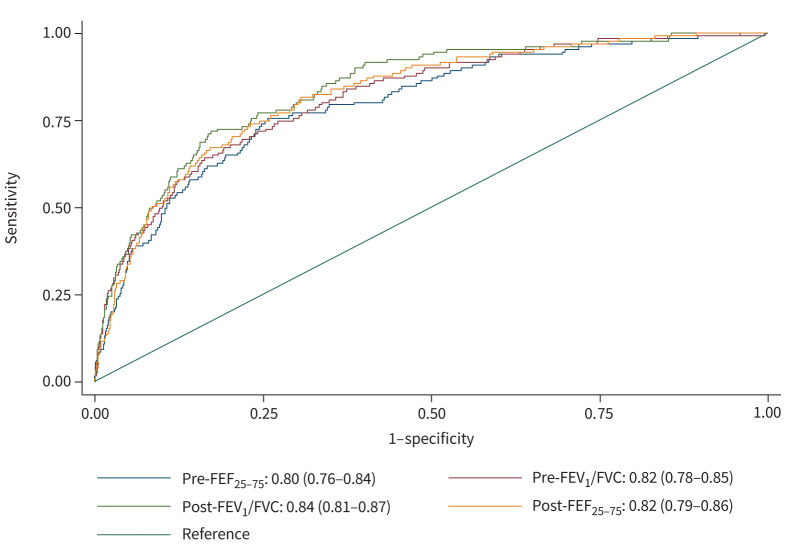
Comparison of the area under the curve (AUC) for pre- and post-bronchodilator spirometry z-scores for incidence of chronic airflow obstruction. FEV_1_/FVC: forced expiratory volume in 1 s as a ratio of the forced vital capacity; FEF_25–75_: mean forced expiratory flow rate between 25% and 75% of the forced vital capacity.

**FIGURE 3:**
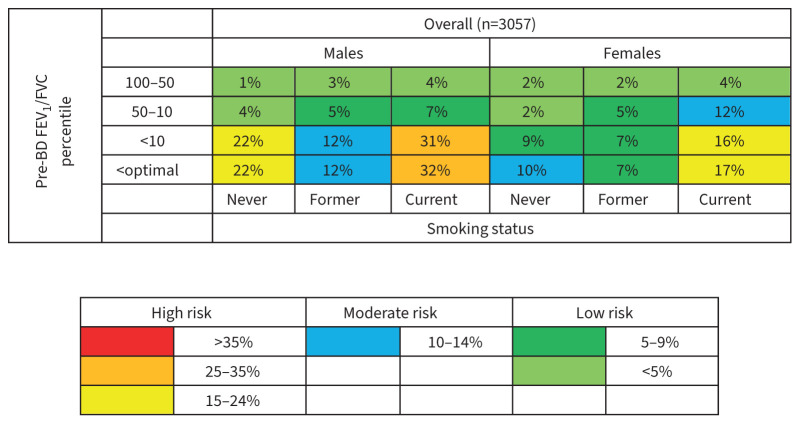
Absolute risk of chronic airflow obstruction incidence stratified by baseline pre-bronchodilator (BD) FEV_1_/FVC percentile, sex and smoking status. Risks categories stratified as per Tan *et al.* [6]. <optimal: less than the optimal pre-BD FEV_1_/FVC z-score threshold of −1.336 (∼ 9th percentile) identified by this study. Absolute risk: number with chronic airflow obstruction in the exposed group divided by the number in the exposed group. FEV_1_/FVC: forced expiratory volume in 1 s to forced vital capacity ratio.

The accuracy of baseline spirometry z-scores to discriminate chronic airflow obstruction incidence was similar for both pre- and post-bronchodilator measurements, with an AUC of between 0.80 and 0.84 (figure 2). Table 2 displays the optimal spirometry thresholds derived from these curves. The highest Youden index was 0.50. This was achieved by pre-bronchodilator FEV_1_/FVC at a z-score of −1.336, which corresponds to the 9th percentile. The sensitivity of the optimal threshold was 78%, specificity 72%, positive likelihood ratio 2.79, and negative likelihood ratio 0.31. The absolute risk of chronic airflow obstruction incidence stratified by pre-bronchodilator FEV_1_/FVC percentile, sex and smoking status is displayed in figure 3. Individuals below the 9/10th percentile had a moderate–high absolute risk of chronic airflow obstruction incidence compared to those above the 9/10th percentile, who had a low–moderate absolute risk. The highest absolute risk was seen in current smokers below the optimal threshold for males (32%) and females (17%). The risk was also high for male never-smokers with a pre-bronchodilator FEV_1_/FVC below the 9th/10th percentile. Post-bronchodilator FEF_25–75_ also achieved a maximum Youden index of 0.50 at a z-score of −1.069, equivalent to the 14th percentile. Sensitivity was 81%, specificity 69%, positive likelihood ratio 2.61 and negative likelihood ratio 0.28. The characteristics of those above and below the optimal thresholds are displayed in table 3. Generally, those below the optimal thresholds for FEV_1_/FVC were more likely female, to be symptomatic and to have ever-smoked than those above the optimal thresholds. The European region had the highest proportion below the optimal FEV_1_/FVC thresholds. When using the GLI Caucasian reference equations, we found that the optimal z-score thresholds were similar to those of the main analysis, with pre-bronchodilator FEV_1_/FVC <8th percentile performing best (supplementary eTable 1). The accuracy of respiratory symptoms to discriminate incidence of chronic airflow obstruction was lower than that of all spirometry thresholds, with the Youden index ranging from 0.30 for chronic cough to 0.37 for chronic phlegm (table 4).

**TABLE 2:** Optimal thresholds for spirometry to predict onset of chronic airflow obstruction

	z-score threshold	Percentile	Sensitivity %	Specificity %	LR^+^	LR^−^	Y_max_	Brier score
**Pre-BD FEV_1_/FVC**	−1.336	<9th	78	72	2.79	0.31	0.50	0.0376
**Post-BD FEV_1_/FVC**	−0.606	<27th	74	75	2.96	0.35	0.49	0.0373
**Pre-BD FEF_25–75_**	−1.453	<7th	72	71	2.48	0.39	0.43	0.0372
**Post-BD FEF_25–75_**	−1.069	<14th	81	69	2.61	0.28	0.50	0.0377

Total participants n=3057. Lower z-scores indicate more severe disease. Optimal thresholds determined by the unweighted Youden index. Z scores calculated using the Rspiro package in R based on reference equations for European Americans in The Third National Health and Nutrition Survey (NHANES III) [14]. Brier score ranges between 0 and 1, with 0 indicating a perfect prediction and 1 a non-informative predictive model [23]. LR: likelihood ratio; Y_max_: Youden index at optimal threshold; BD: bronchodilator (200 µg salbutamol); FEV_1_/FVC: forced expiratory volume in 1 s as a ratio of the forced vital capacity; FEF_25–75_: mean forced expiratory flow rate between 25% and 75% of the forced vital capacity.

**TABLE 3:** Baseline characteristics of participants above and below the optimal thresholds

	Above all thresholds	Pre-BD FEV_1_/FVC z-score <−1.336	Post-BD FEV_1_/FVC z-score <−0.606	Pre-BD FEF_25–75_ z-score <−1.453	Post-BD FEF_25–75_ z-score <−1.069
**Participants, n**	1945	371	540	737	763
**Age years, mean±sd**	51.8±9.2	52.1±9.1	52.4±9.5	49.1±7.4	49.5±7.7
**Female, n (%)**	1032 (53)	230 (62)	318 (59)	480 (65)	485 (64)
**Follow-up time, years, median (IQR)**	8.4 (6.1–11.0)	9.7 (6.2–12.9)	8.3 (6.1–11.5)	8.2 (6.1–10.8)	8.2 (6.2–10.8)
**Smoking status, n (%)**					
Never	1403 (72)	237 (64)	340 (63)	582 (79)	595 (78)
Former	274 (14)	68 (18)	93 (17)	59 (8)	65 (9)
Current	268 (14)	66 (18)	107 (20)	96 (13)	103 (13)
**Pack years, mean±sd**	5.0±17.4	6.2±12.2	8.3±37.4	3.6±10.1	4.1±10.9
**Symptoms, n (%)**					
Dyspnoea	259 (13)	63 (17)	90 (17)	107 (14)	104 (14)
Chronic cough	70 (4)	27 (7)	29 (5)	26 (3)	27 (4)
Phlegm	67 (3)	22 (6)	27 (5)	30 (4)	30 (4)
Wheeze	187 (10)	61 (16)	78 (14)	82 (11)	81 (11)
At least one symptom	437 (22)	123 (33)	170 (32)	183 (25)	179 (23)
**Spirometry, mean±sd**					
Pre-BD FEV_1_/FVC z-score	0.21±0.72	−1.79±0.39	−1.30±0.71	−1.10±0.84	−0.85±0.85
Post-BD FEV_1_/FVC z-score	0.61±0.64	−0.86±0.51	−1.03±0.29	−0.44±0.70	−0.57±0.61
Pre-BD FEF_25–75_ z-score	−0.20±0.85	−1.89±0.56	−1.58±0.65	−1.94±0.40	−1.75±0.58
Post-BD FEF_25–75_ z-score	0.26±0.86	−1.30±0.61	−1.39±0.61	−1.41±0.62	−1.58±0.40
**WHO world region, n (%)**					
Africa	362 (19)	79 (21)	114 (21)	179 (24)	174 (23)
Americas	17 (1)	0 (0)	1 (0)	2 (0)	0 (0)
South-East Asia	417 (21)	58 (16)	97 (18)	266 (36)	291 (38)
Europe	821 (42)	185 (50)	255 (47)	160 (22)	156 (20)
Eastern Mediterranean	186 (10)	18 (5)	39 (7)	66 (9)	75 (10)
Western Pacific	142 (7)	31 (8)	34 (6)	64 (9)	67 (9)

Dyspnoea measured according to mMRC dyspnoea scale: 0–1=minimal/no breathlessness; ≥2=significant breathlessness. Chronic cough: cough on most days for 3 months each year. Chronic phlegm: phlegm on most days 3 months each year. Wheeze: wheezing or whistling in the chest at any time in the last 12 months. z-scores calculated using the Rspiro package in R based on reference equations for European Americans in The Third National Health and Nutrition Survey (NHANES III) [14]. BD: bronchodilator (200 µg salbutamol); FEV_1_/FVC: forced expiratory volume in 1 s as a ratio of the forced vital capacity; FEF_25–75_: mean forced expiratory flow rate between 25% and 75% of the forced vital capacity; WHO: World Health Organization.

**TABLE 4:** Accuracy of respiratory symptoms to discriminate incidence of chronic airflow obstruction

	n	CAO (follow-up), n (%)	Sensitivity %	Specificity %	LR^+^	LR^−^	Youden index
**Dyspnoea**	418	26 (6)	72	59	1.76	0.47	0.31
**Chronic cough**	113	11 (10)	66	64	1.83	0.53	0.30
**Chronic phlegm**	113	12 (11)	66	71	2.28	0.48	0.37
**Wheeze**	309	33 (11)	68	63	1.84	0.51	0.32
**At least one symptom**	713	55 (8)	63	70	2.10	0.53	0.33

Dyspnoea measured according to mMRC dyspnoea scale: 0–1=minimal/no breathlessness; ≥2=significant breathlessness. Chronic cough: cough on most days for 3 months each year. Chronic phlegm: phlegm on most days 3 months each year. Wheeze: wheezing or whistling in the chest at any time in the last 12 months. At follow-up, chronic airflow obstruction (CAO) was diagnosed if the post-BD (200 µg salbutamol) FEV_1_/FVC was <LLN. LLN calculated using reference equations for European Americans from the NHANES III study population [14]. LR: likelihood ratio.

Table 5 summarises the association of the optimal spirometry thresholds with incident chronic airflow obstruction. 371 participants (12%) were below the optimal FEV_1_/FVC z-score threshold of −1.336 at baseline. Of those, 54 (16%) developed chronic airflow obstruction at follow-up. This was associated with having a significantly lower post-bronchodilator FEV_1_/FVC (β: −5.88%, 95% CI −7.31 to −4.43) and a 4.9-fold increase in odds of developing chronic airflow obstruction at follow-up (OR: 4.89, 95% CI 2.32 to 9.33). Similar results were seen for the other three spirometry thresholds. There was no significant interaction between smoking status and being below the optimal spirometry thresholds on the incidence of chronic airflow obstruction. The Brier scores for the optimal thresholds were similar, ranging from 0.0372 to 0.0377 (table 2), suggesting acceptable overall model performance. When comparing the association between different percentile thresholds for pre-bronchodilator FEV_1_/FVC and incidence of chronic airflow obstruction, the optimal threshold identified in this study performed best; however, the 95% confidence intervals were overlapping from the 7th to the 25th percentile, suggesting they were statistically similar (supplementary eFigure 1). Adding a history of at least one respiratory symptom at baseline as a covariate did not improve the ability of the thresholds to discriminate incident chronic airflow obstruction (supplementary efigure 2).

**TABLE 5:** Association of optimal thresholds and respiratory symptoms with incidence of chronic airflow obstruction

	n	CAO (follow-up), n (%)	OR (95% CI)	p-value	p-value for interaction with smoking status (ever/never)	β coefficient (95% CI)^#^	p-value
**Pre-BD FEV_1_/FVC z-score <−1.336**	371	54 (16)	4.89 (2.32–9.33)	<0.0001	0.0637	−5.88 (−7.31– −4.43)	<0.0001
**Post-BD FEV_1_/FVC z-score <−0.606**	540	75 (14)	5.28 (2.64–10.54)	<0.0001	0.8209	−5.75 (−7.09– −4.41)	<0.0001
**Pre-BD FEF_25–75_ z-score <−1.453**	737	67 (9)	4.20 (1.51–11.7)	0.006	0.0896	−4.36 (−5.81– −2.90)	<0.0001
**Post-BD FEF_25–75_ z-score <−1.069**	763	69 (9)	5.07 (2.62–9.79)	<0.0001	0.7252	−5.52 (−6.80– −4.23)	<0.0001
**At least one respiratory symptom**	713	55 (8)	1.89 (1.21–2.96)	0.005	0.1429	−1.32 (−2.15– −0.50)	0.002

Total participants n=3057. Linear associations between being below an optimal threshold or having at least one respiratory symptom and follow-up post-bronchodilator FEV_1_/FVC ratio were estimated using mixed effects linear regression models. Associations between being below an optimal threshold or having at least one respiratory symptom and progression to chronic airflow obstruction (CAO) were estimated using mixed effects logistic regression models. Models were adjusted for sex, age, BMI, smoking status, smoking pack-years and FVC. As we expected associations to vary by study site, we fitted a random intercept to account for clustering by site and a random slope to average the associations across study sites. Optimal thresholds classified according to z-score cut-offs displayed in the table. z-scores calculated using the Rspiro package in R based on reference equations for European Americans in The Third National Health and Nutrition Survey (NHANES III) [14]. At follow-up, CAO was diagnosed if the post-BD (200 µg salbutamol) FEV_1_/FVC was <LLN. LLN calculated using reference equations from the NHANES III study population [14]. BD: bronchodilator; FEV_1_/FVC: forced expiratory volume in 1 s as a ratio of the forced vital capacity; FEF_25–75_: mean forced expiratory flow rate between 25% and 75% of the forced vital capacity. ^#^: negative regression coefficient indicates a reduction in FEV_1_/FVC ratio (*i.e.*, worsened lung function).

713 participants (23%) reported at least one respiratory symptom at baseline, of which 55 (8%) developed chronic airflow obstruction at follow-up. Having at least one respiratory symptom was associated with a lower post-bronchodilator FEV_1_/FVC (β: −1.32%, 95% CI −2.15 to −0.50) and greater odds of chronic airflow obstruction at follow-up (OR: 1.89, 95% CI 1.21 to 2.96). The association was of a smaller magnitude to that seen for the optimal spirometry thresholds.

Supplementary eFigure 3 compares the discriminative ability of the optimal spirometry thresholds identified by this study to those of Tan *et al.* [6] and Kwon *et al.* [7]. The z-score thresholds performed similarly in discriminating incident chronic airflow obstruction, with the AUCs for pre-bronchodilator thresholds ranging from 0.76 to 0.79 and post-bronchodilator thresholds from 0.77 to 0.82.

In the present study, agreement between the optimal thresholds was moderate (supplementary efigure 4), with 59% concordance between the optimal pre-bronchodilator FEV_1_/FVC and FEF_25–75_ thresholds and 62% between the post-bronchodilator thresholds in discriminating incident chronic airflow obstruction at follow-up.

## Discussion

We have shown that spirometry parameters are good at discriminating chronic airflow obstruction incidence, while respiratory symptoms perform poorly in comparison. We have identified optimal z-score thresholds for FEV_1_/FVC and FEF_25–75_, which are consistent with those identified by Tan *et al.* [6].

We found that the highest Youden index was achieved for pre-bronchodilator FEV_1_/FVC z-score <−1.336, which is equivalent to the 9th percentile of a normal non-smoking population. This is very similar to findings by Tan *et al.* [6], who found that a pre-bronchodilator FEV_1_/FVC z-score equivalent to the 10th percentile best discriminated chronic airflow obstruction incidence. The threshold we identified for post-bronchodilator FEV_1_/FVC was also similar, with ours equivalent to the 27th percentile and theirs the 24th. While our results externally validate those of Tan *et al.* [6], we universally found a lower Youden index, sensitivity and specificity. A potential reason for this is the heterogeneous nature of the BOLD study. It has previously been shown that the prevalence of chronic airflow obstruction varies greatly across BOLD study sites, as does the prevalence of risk factors such as tobacco smoking [3]. In comparison, the Tasmanian Longitudinal Health Study has a more homogeneous population than BOLD, where the appropriateness of the optimal thresholds may vary across study sites. Furthermore, we used Caucasian reference equations for all study sites, the rationale being that the prevalence of airflow obstruction using the NHANES reference equations has previously been shown to be similar regardless of whether race is accounted for. However, it is possible this approach reduces the sensitivity and specificity of our models in comparison to those of Tan *et al*. Taken together, our results suggest that the current LLN for FEV_1_/FVC, which is equivalent to the 5th percentile, may fail to identify all those with disease. In particular, consideration should be given to people who fall between the 5th and 10th percentile for FEV_1_/FVC, which may reflect early airflow obstruction or pre-COPD. It is important that future studies compare the appropriateness of these new thresholds in comparison to the LLN in different populations, as due to sample size limitations, we were unable to stratify our analyses at the site level.

We found that respiratory symptoms performed poorly in comparison to spirometry for discriminating chronic airflow obstruction incidence, with chronic phlegm having the highest Youden index. Despite this, those with at least one respiratory symptom were approximately twice as likely to progress to chronic airflow obstruction than those without respiratory symptoms. Our results are similar to those of Ohar *et al.* [25], who found that respiratory symptoms were associated with a 1.2- to 2-fold increase in the odds of chronic airflow obstruction, but that they conveyed no additional benefit over age and smoking status to the predictive power of spirometry. Their study was in a population referred for work-related medical evaluation, as such, the prevalence of respiratory symptoms was very high, even among those with normal lung function. A more comparable study is that of Tan *et al.* [6], who also demonstrated that respiratory symptoms poorly discriminated chronic airflow obstruction incidence in comparison to spirometry in a population-based cohort study. Together, our results suggest that while respiratory symptoms alone are a risk factor for future chronic airflow obstruction, spirometry has better predictive ability and should be used in combination with respiratory symptoms to inform diagnosis and risk stratification.

The clinical relevance of our findings relates to the external validation of the thresholds identified by Tan *et al.* [6] in a separate population-based cohort study. Of particular importance is our joint finding that a pre-bronchodilator FEV_1_/FVC equivalent to the 9th/10th percentile represents the optimal threshold for predicting chronic airflow obstruction incidence. While it is not entirely surprising that a low–normal FEV_1_/FVC is associated with increased odds of chronic airflow obstruction, it provides confirmation of a framework that could be utilised to define early airflow obstruction or pre-COPD. We also identified that the absolute risk of progression to chronic airflow obstruction was highest among current smokers below the 9th percentile. However, the interaction was not significant in the adjusted model, and never-smokers also had a high absolute risk, suggesting that this threshold is appropriate regardless of smoking history. Furthermore, as this is a pre-bronchodilator threshold, it is easier to implement in primary care and low resource settings where post-bronchodilator spirometry is seldom performed. We also found that FEF_25–75_ and FEV_1_/FVC identified slightly different at-risk populations, suggesting that the parameters capture slightly different physiological characteristics.

Our study has several strengths. First, its large sample size and population-based design make the results transferable to general populations. Spirometry was conducted by trained and certified technicians and lung function data were quality assured centrally. We also used z-scores and the LLN to define abnormal results, which are widely accepted to be more appropriate than per cent predicted cut-offs, which are prone to misclassification [26]. Our study also has limitations. The longitudinal component of this study was impacted by significant loss to follow-up caused by the COVID-19 pandemic. Although we attempted to account for this by using inverse probability weights, it is possible that those present at follow-up are not entirely representative of the general population, which will influence the generalisability of the results. Our study is also not generalisable to those below the age of 40 or above the age of 60, as we did not have data on individuals in this age range. We were also limited by sample size at site level, especially where prevalence of chronic airflow obstruction at follow-up was low, which restricted our ability to perform stratified analyses by study site. Furthermore, as we only had one timepoint of follow-up spirometry, it is possible that some intra-individual variability in spirometry measurements were responsible for differences in lung function over time. Finally, as follow-up periods varied considerably, it is possible that for some sites follow-up duration was insufficient for people below the optimal thresholds to develop chronic airflow obstruction.

### Conclusions

In conclusion, we have shown that spirometry is a better predictor of chronic airflow obstruction incidence than respiratory symptoms alone. We have externally validated the optimal z-score thresholds identified by Tan *et al.* [6] and confirmed that a pre-bronchodilator FEV_1_/FVC <10th percentile could suggest early airflow obstruction or pre-COPD. Future studies should replicate our findings using larger sample sizes in specific populations across the globe.

## Supplementary material

10.1183/23120541.00624-2024.Supp1 **Please note:** supplementary material is not edited by the Editorial Office, and is uploaded as it has been supplied by the author.
Supplementary material 00674-2024.SUPPLEMENT
