# Supplementary material for: Optimal spirometry thresholds for the prediction of chronic airflow obstruction: a multinational longitudinal study
Source: ERJ Open Res. 2025 Mar 3;11(2):00624-2024. doi: 10.1183/23120541.00624-2024 (PMC11873882; doi:10.1183/23120541.00624-2024)
Supplement: Supplementary file 1 [file 00674-2024.SUPPLEMENT.pdf]

Supplementary Table 1. *Correlation matrix of the natural logarithm of blood biomarkers levels in the included study population (n=10602).*

	**CRP (ln)**	**B-Eos (ln)**	**B-Neu (ln)**	**B-Lym (ln)**	**B-Leu (ln)**	**B-Mono (ln)**
	PCC (95% CI)	PCC (95% CI)	PCC (95% CI)	PCC (95% CI)	PCC (95% CI)	PCC (95% CI)
Blood biomarkers						
CRP (ln)	**1.00 (1.00, 1.00)**	-	-	-	-	-
B-Eos (ln)	**0.12 (0.10, 0.14)**	**1.00 (1.00, 1.00)**	-	-	-	-
B-Neu (ln)	**0.33 (0.32, 0.35)**	**0.17 (0.15, 0.18)**	**1.00 (1.00, 1.00)**	-	-	-
B-Lym (ln)	**0.11 (0.10, 0.13)**	**0.23 (0.21, 0.24)**	**0.22 (0.21, 0.24)**	**1.00 (1.00, 1.00)**	-	-
B-Leu (ln)	**0.33 (0.31, 0.34)**	**0.31 (0.29, 0.33)**	**0.88 (0.87, 0.88)**	**0.62 (0.61, 0.63)**	**1.00 (1.00, 1.00)**	-
B-Mono (ln)	**0.25 (0.24, 0.27)**	**0.25 (0.24, 0.27)**	**0.51 (0.50, 0.53)**	**0.35 (0.33, 0.36)**	**0.63 (0.61, 0.64)**	**1.00 (1.00, 1.00)**

* Pearson's correlation test. Results were statistically significant if the 95% CI does not include 0 (shown in bold)

B-Eos: blood eosinophils, B-Neu: blood neutrophils, B-Leu: blood leukocytes, B-Lym: blood lymphocytes, B-Mono: blood monocytes, CI: confidence interval, CRP: C-reactive protein ln: natural logarithm, PCC: Pearson correlation coefficient

***Supplementary Table 2**. Crude logistic regression models of blood biomarker levels in tertiles and abnormal impulse oscillometry indices (n=10602).*

	**Abnormal R_5 1) (n=722)**	**Abnormal R_{20} 1) (n=629)**	**Abnormal X_5 2) (n=777)**
	OR (95% CI)	OR (95% CI)	OR (95% CI)
***Blood biomarkers* 4)**			
CRP, tertile 2 vs 1	**1.28 (1.06, 1.56)**	**1.30 (1.05, 1.59)**	**1.25 (1.02, 1.52)**
CRP, tertile 3 vs 1	**1.63 (1.35, 1.97)**	**1.57 (1.28, 1.92)**	**2.14 (1.78, 2.57)**
B-Eos, tertile 2 vs 1	1.07 (0.90, 1.28)	1.03 (0.86, 1.24)	1.09 (0.91, 1.29)
B-Eos, tertile 3 vs 1	**1.22 (1.01, 1.48)**	0.96 (0.78, 1.19)	**1.61 (1.35, 1.92)**
B-Neu, tertile 2 vs 1	1.12 (0.92, 1.36)	1.05 (0.85, 1.29)	1.12 (0.92, 1.35)
B-Neu, tertile 3 vs 1	**1.60 (1.33, 1.93)**	**1.47 (1.21, 1.79)**	**1.74 (1.45, 2.09)**
B-Lym, tertile 2 vs 1	0.96 (0.79, 1.17)	0.89 (0.73, 1.10)	1.13 (0.93, 1.36)
B-Lym, tertile 3 vs 1	**1.41 (1.18, 1.68)**	1.17 (0.97, 1.40)	**1.59 (1.34, 1.89)**
B-Leu, tertile 2 vs 1	1.16 (0.96, 1.41)	1.13 (0.92, 1.39)	**1.28 (1.05, 1.55)**
B-Leu, tertile 3 vs 1	**1.61 (1.34, 1.94)**	**1.44 (1.18, 1.75)**	**1.91 (1.59, 2.30)**
B-Mono, tertile 2 vs 1	**1.26 (1.04, 1.52)**	**1.28 (1.05, 1.56)**	**1.24 (1.03, 1.50)**
B-Mono, tertile 3 vs 1	**1.52 (1.27, 1.81)**	**1.46 (1.21, 1.76)**	**1.68 (1.41, 1.98)**

	**Abnormal AX 1) (n=790)**	**Abnormal f_{res} 1) (n=746)**	**Any abnormal IOS 3) (n=1715)**
	OR (95% CI)	OR (95% CI)	OR (95% CI)
CRP, tertile 2 vs 1	**1.29 (1.06, 1.56)**	1.21 (0.99, 1.46)	**1.22 (1.07, 1.40)**
CRP, tertile 3 vs 1	**1.90 (1.58, 2.28)**	**1.62 (1.34, 1.94)**	**1.73 (1.52, 1.97)**
B-Eos, tertile 2 vs 1	1.13 (0.95, 1.33)	1.14 (0.96, 1.36)	1.11 (0.98, 1.25)
B-Eos, tertile 3 vs 1	**1.41 (1.18, 1.69)**	**1.39 (1.16, 1.68)**	**1.30 (1.14, 1.49)**
B-Neu, tertile 2 vs 1	1.11 (0.92, 1.34)	**1.28 (1.06, 1.55)**	**1.16 (1.01, 1.32)**
B-Neu, tertile 3 vs 1	**1.57 (1.31, 1.88)**	**1.62 (1.34, 1.95)**	**1.57 (1.38, 1.78)**
B-Lym, tertile 2 vs 1	1.17 (0.97, 1.41)	1.17 (0.97, 1.42)	1.09 (0.95, 1.24)
B-Lym, tertile 3 vs 1	**1.67 (1.41, 1.98)**	**1.61 (1.35, 1.92)**	**1.40 (1.24, 1.58)**
B-Leu, tertile 2 vs 1	1.11 (0.92, 1.34)	1.18 (0.97, 1.43)	**1.15 (1.01, 1.32)**
B-Leu, tertile 3 vs 1	**1.65 (1.38, 1.98)**	**1.67 (1.39, 2.01)**	**1.58 (1.39, 1.79)**
B-Mono, tertile 2 vs 1	**1.26 (1.05, 1.51)**	**1.17 (0.97, 1.41)**	**1.22 (1.07, 1.39)**
B-Mono, tertile 3 vs 1	**1.47 (1.24, 1.75)**	**1.42 (1.19, 1.69)**	**1.49 (1.32, 1.68)**

* Results were statistically significant if the 95% CI does not include 1 (shown in bold)
1) Abnormal R_5, R_{20}, AX, f_{res} were defined as indices above the ULN (>95th percentile)
2) Abnormal X_5 was defined as indices below the LLN (<5th percentile)
3) Any abnormal IOS was defined as having either abnormal R_5, R_{20}, AX, f_{res} above the ULN (>95th percentile) and/or abnormal X_5 below the LLN (<5th percentile)
4) The 1st, 2nd and 3rd tertile corresponded to a level of 0.20-0.73 (n=3566), 0.74-1.80 (n=3559) and 1.90-127 (n=3477) mg/L [CRP], 0.03-0.10 (n=5048), 0.20-0.20 (n=2265) and 0.30-2.10 (n=3289) 10^9/L [B-Eos], 0.70-2.50 (n=3566), 2.60-3.40 (n=3767) and 3.50-12.9 (n=3269) 10^9/L [B-Neu], 0.30-1.60 (n=4262), 1.70-2.00 (n=3019) and 2.10-64.5 (n=3321) 10^9/L [B-Lym], 2.10-4.90 (n=3614), 5.00-6.10 (n=3590) and 6.20-87.3 (n=3398) 10^9/L [B-Leu], and 0.10-0.40 (n=5124), 0.50-0.50 (n=2636) and 0.60-1.70 (n=2842) 10^9/L [B-Mono], respectively

AX: area of reactance, B-Eos: blood eosinophils, B-Neu: blood neutrophils, B-Leu: blood leukocytes, B-Lym: blood lymphocytes, B-Mono: blood monocytes, BMI: body mass index, CI: confidence interval, CRP: C-reactive protein, f_{res}: resonance frequency, Hz: hertz, IOS: impulse oscillometry, L: litre, LLN: lower limit of normal, mg/L: milligrams per litre, n: number, OR: odds ratio, R_5: resistance at 5 Hz, R_{20}: resistance at 20 Hz, ULN: upper limit of normal, X_5: reactance at 5 Hz

Supplementary Table 3*. Crude logistic regression models of number of blood biomarker in the upper tertile and abnormal impulse oscillometry indices (n=10602).*

	**Abnormal R_5 1) (n=722)**	**Abnormal R_{20} 1) (n=629)**	**Abnormal X_5 2) (n=777)**
	OR (95% CI)	OR (95% CI)	OR (95% CI)
***Numbers of blood biomarkers in upper tertile* 4)**			
1	1.15 (0.93, 1.44)	1.04 (0.83, 1.31)	**1.50 (1.20, 1.87)**
2-3	1.12 (0.90, 1.39)	1.09 (0.87, 1.36)	**1.56 (1.26, 1.94)**
4-6	**2.02 (1.64, 2.48)**	**1.61 (1.29, 2.01)**	**2.86 (2.23. 3.53)**

	**Abnormal AX 1) (n=790)**	**Abnormal f_{res} 1) (n=746)**	**Any abnormal IOS 3) (n=1715)**
	OR (95% CI)	OR (95% CI)	OR (95% CI)
1	**1.43 (1.15, 1.77)**	**1.40 (1.13, 1.75)**	**1.24 (1.07, 1.44)**
2-3	**1.52 (1.23, 1.88)**	**1.46 (1.18, 1.81)**	**1.33 (1.15, 1.53)**
4-6	**2.43 (1.98, 2.99)**	**2.20 (1.78, 2.72)**	**2.04 (1.76, 2.36)**

* Results were statistically significant if the 95% CI does not include 1 (shown in bold)
1) Abnormal R_5, R_{20}, AX, f_{res} were defined as indices above the ULN (>95th percentile)
2) Abnormal X_5 was defined as indices below the LLN (<5th percentile)
3) Any abnormal IOS was defined as having either abnormal R_5, R_{20}, AX, f_{res} above the ULN (>95th percentile) and/or abnormal X_5 below the LLN (<5th percentile)
4) 1, 2-3 and 4-6 blood biomarkers in the upper tertile were defined as having 1, 2-3 and 4-6 biomarkers (CRP, B-Eos, B-Neu, B-Lym, B-Leu, B-Mono) in the upper tertile, respectively

AX: area of reactance, B-Eos: blood eosinophils, B-Neu: blood neutrophils, B-Leu: blood leukocytes, B-Lym: blood lymphocytes, B-Mono: blood monocytes, BMI: body mass index, CI: confidence interval, CRP: C-reactive protein, f_{res}: resonance frequency, Hz: hertz, IOS: impulse oscillometry, LLN: lower limit of normal, n: number, OR: odds ratio, R_5: resistance at 5 Hz, R_{20}: resistance at 20 Hz, ULN: upper limit of normal, X_5: reactance at 5 Hz

Supplementary Table 4. *Crude (**A**) and adjusted (**B**) logistic regression models of blood biomarker levels in tertiles and abnormal impulse oscillometry indices in participants with normal spirometry (n=8853).*

A.

	**Abnormal R_5 [1)] (n=486)**	**Abnormal R_{20} [1)] (n=456)**	**Abnormal X_5 [2)] (n=434)**
	OR (95% CI)	OR (95% CI)	OR (95% CI)
Blood biomarkers **[4)]**			
CRP, tertile 2 vs 1	**1.28 (1.02, 1.61)**	1.23 (0.98, 1.56)	1.13 (0.89, 1.45)
CRP, tertile 3 vs 1	**1.34 (1.06, 1.68)**	**1.36 (1.08, 1.72)**	**1.49 (1.17, 1.89)**
B-Eos, tertile 2 vs 1	1.16 (0.94, 1.42)	1.05 (0.85, 1.29)	1.05 (0.84, 1.31)
B-Eos, tertile 3 vs 1	1.07 (0.84, 1.37)	0.87 (0.67, 1.13)	1.27 (0.99, 1.62)
B-Neu, tertile 2 vs 1	0.96 (0.76, 1.20)	0.97 (0.77, 1.23)	0.95 (0.75, 1.21)
B-Neu, tertile 3 vs 1	**1.31 (1.05, 1.64)**	**1.26 (1.01, 1.59)**	**1.31 (1.04, 1.65)**
B-Lym, tertile 2 vs 1	0.91 (0.72, 1.15)	0.80 (0.63, 1.01)	1.07 (0.83, 1.37)
B-Lym, tertile 3 vs 1	**1.26 (1.02, 1.56)**	1.00 (0.81, 1.25)	**1.51 (1.21, 1.90)**
B-Leu, tertile 2 vs 1	1.01 (0.81, 1.28)	1.04 (0.83, 1.31)	1.18 (0.93, 1.50)
B-Leu, tertile 3 vs 1	**1.39 (1.11, 1.73)**	1.20 (0.96, 1.52)	**1.51 (1.19, 1.92)**
B-Mono, tertile 2 vs 1	1.18 (0.94, 1.48)	1.17 (0.93, 1.47)	1.26 (0.99, 1.60)
B-Mono, tertile 3 vs 1	**1.25 (1.01, 1.56)**	1.21 (0.96, 1.52)	**1.44 (1.14, 1.81)**

	**Abnormal A*X* [1)] (n=455)**	**Abnormal f_{res} [1)] (n=436)**	**Any abnormal IOS [3)] (n=1135)**
	OR (95% CI)	OR (95% CI)	OR (95% CI)
CRP, tertile 2 vs 1	1.26 (0.99, 1.60)	1.21 (0.96, 1.54)	**1.18 (1.01, 1.37)**
CRP, tertile 3 vs 1	**1.48 (1.17, 1.88)**	**1.29 (1.02, 1.64)**	**1.43 (1.23, 1.67)**
B-Eos, tertile 2 vs 1	1.17 (0.94, 1.45)	1.15 (0.92, 1.43)	1.10 (0.96, 1.27)
B-Eos, tertile 3 vs 1	1.23 (0.96, 1.57)	**1.29 (1.01, 1.65)**	1.13 (0.96, 1.33)
B-Neu, tertile 2 vs 1	0.96 (0.76, 1.21)	1.09 (0.86, 1.38)	1.04 (0.89, 1.21)
B-Neu, tertile 3 vs 1	1.21 (0.96, 1.53)	**1.29 (1.02, 1.64)**	**1.28 (1.10, 1.50)**
B-Lym, tertile 2 vs 1	1.14 (0.89, 1.45)	1.15 (0.90, 1.46)	0.98 (0.84, 1.15)
B-Lym, tertile 3 vs 1	**1.52 (1.22, 1.91)**	**1.46 (1.16, 1.83)**	**1.25 (1.08, 1.45)**
B-Leu, tertile 2 vs 1	0.95 (0.75, 1.20)	1.01 (0.79, 1.29)	1.05 (0.91, 1.23)
B-Leu, tertile 3 vs 1	**1.35 (1.08, 1.70)**	**1.44 (1.15, 1.82)**	**1.30 (1.11, 1.51)**
B-Mono, tertile 2 vs 1	1.23 (0.97, 1.56)	1.11 (0.88, 1.42)	**1.19 (1.02, 1.38)**
B-Mono, tertile 3 vs 1	**1.36 (1.08, 1.70)**	**1.30 (1.04, 1.64)**	**1.31 (1.12, 1.52)**

***B*.**

	**Abnormal R_5 1)** (n=486)	**Abnormal R_{20} 1)** (n=456)	**Abnormal X_5 2)** (n=434)
	OR (95% CI)	OR (95% CI)	OR (95% CI)
***Blood biomarkers* 4)**			
CRP, tertile 2 vs 1	1.25 (0.98, 1.58)	1.22 (0.96, 1.56)	1.10 (0.85, 1.41)
CRP, tertile 3 vs 1	**1.35 (1.04, 1.75)**	**1.42 (1.09, 1.86)**	1.22 (0.93, 1.59)
B-Eos, tertile 2 vs 1	1.11 (0.90, 1.37)	1.01 (0.81, 1.25)	1.01 (0.80, 1.27)
B-Eos, tertile 3 vs 1	0.97 (0.76, 1.25)	0.81 (0.62, 1.06)	1.19 (0.92, 1.53)
B-Neu, tertile 2 vs 1	0.89 (0.71, 1.12)	0.93 (0.74, 1.18)	0.92 (0.72, 1.17)
B-Neu, tertile 3 vs 1	1.08 (0.85, 1.38)	1.11 (0.86, 1.42)	1.14 (0.88, 1.47)
B-Lym, tertile 2 vs 1	0.87 (0.69, 1.10)	0.77 (0.61, 0.98)	1.03 (0.80, 1.32)
B-Lym, tertile 3 vs 1	1.16 (0.93, 1.44)	0.93 (0.74, 1.17)	**1.36 (1.08, 1.72)**
B-Leu, tertile 2 vs 1	0.94 (0.75, 1.19)	0.99 (0.78, 1.26)	1.14 (0.89, 1.46)
B-Leu, tertile 3 vs 1	1.22 (0.96, 1.55)	1.08 (0.84, 1.39)	**1.38 (1.07, 1.79)**
B-Mono, tertile 2 vs 1	1.12 (0.89, 1.40)	1.13 (0.89, 1.43)	1.23 (0.96, 1.57)
B-Mono, tertile 3 vs 1	1.06 (0.84, 1.35)	1.09 (0.85, 1.39)	**1.37 (1.07, 1.75)**

	**Abnormal AX 1)** (n=455)	**Abnormal f_{res} 1)** (n=436)	**Any abnormal IOS 3)** (n=1135)
	OR (95% CI)	OR (95% CI)	OR (95% CI)
CRP, tertile 2 vs 1	1.14 (0.89, 1.46)	1.19 (0.93, 1.52)	1.15 (0.98, 1.35)
CRP, tertile 3 vs 1	**1.35 (1.03, 1.77)**	**1.37 (1.04, 1.79)**	**1.38 (1.16, 1.64)**
B-Eos, tertile 2 vs 1	1.13 (0.90, 1.41)	1.13 (0.90, 1.41)	1.07 (0.92, 1.23)
B-Eos, tertile 3 vs 1	1.17 (0.91, 1.51)	1.26 (0.98, 1.62)	1.07 (0.91, 1.26)
B-Neu, tertile 2 vs 1	0.93 (0.73, 1.18)	1.10 (0.86, 1.40)	1.01 (0.86, 1.17)
B-Neu, tertile 3 vs 1	1.12 (0.87, 1.44)	1.27 (0.98, 1.65)	1.16 (0.98, 1.37)
B-Lym, tertile 2 vs 1	1.08 (0.85, 1.38)	1.13 (0.88, 1.44)	0.95 (0.81, 1.12)
B-Lym, tertile 3 vs 1	**1.44 (1.15, 1.82)**	**1.48 (1.17, 1.87)**	**1.18 (1.01, 1.37)**
B-Leu, tertile 2 vs 1	0.91 (0.71, 1.16)	1.01 (0.79, 1.29)	1.02 (0.87, 1.19)
B-Leu, tertile 3 vs 1	1.27 (0.99, 1.63)	**1.49 (1.15, 1.91)**	**1.20 (1.02, 1.41)**
B-Mono, tertile 2 vs 1	1.23 (0.97, 1.56)	1.11 (0.87, 1.41)	1.15 (0.98, 1.35)
B-Mono, tertile 3 vs 1	**1.29 (1.01, 1.65)**	1.27 (0.99, 1.63)	**1.20 (1.02, 1.41)**

* Adjusted for age, sex, BMI, smoking and study site. Results were statistically significant if the 95% CI does not include 1 (shown in bold)
1) Abnormal R_5, R_{20}, AX, f_{res} were defined as indices above the ULN (>95th percentile)
2) Abnormal X_5 was defined as indices below the LLN (<5th percentile)
3) Any abnormal IOS was defined as having either abnormal R_5, R_{20}, AX, f_{res} above the ULN (>95th percentile) and/or abnormal X_5 below the LLN (<5th percentile)
4) The 1st, 2nd and 3rd tertile corresponded to a level of 0.20-0.73 (n=3566), 0.74-1.80 (n=3559) and 1.90-127 (n=3477) mg/L [CRP], 0.03-0.10 (n=5048), 0.20-0.20 (n=2265) and 0.30-2.10 (n=3289) 10^9/L [B-Eos], 0.70-2.50 (n=3566), 2.60-3.40 (n=3767) and 3.50-12.9 (n=3269) 10^9/L [B-Neu], 0.30-1.60 (n=4262), 1.70-2.00 (n=3019) and 2.10-64.5 (n=3321) 10^9/L [B-Lym], 2.10-4.90 (n=3614), 5.00-6.10 (n=3590) and 6.20-87.3 (n=3398) 10^9/L [B-Leu], and 0.10-0.40 (n=5124), 0.50-0.50 (n=2636) and 0.60-1.70 (n=2842) 10^9/L [B-Mono], respectively

AX: area of reactance, B-Eos: blood eosinophils, B-Neu: blood neutrophils, B-Leu: blood leukocytes, B-Lym: blood lymphocytes, B-Mono: blood monocytes, BMI: body mass index, CI: confidence interval, CRP: C-reactive protein, f_{res}: resonance frequency, Hz: hertz, IOS: impulse oscillometry, L: litre, LLN: lower limit of normal, n: number, mg/L: milligrams per litre, OR: odds ratio, R_5: resistance at 5 Hz, R_{20}: resistance at 20 Hz, ULN: upper limit of normal, X_5: reactance at 5 Hz

Supplementary Table 5*. Crude (A) and adjusted (**B**) logistic regression models number of blood biomarker in the upper tertile and abnormal impulse oscillometry indices in participants with normal spirometry indices (n=8853).*

A.

	**Abnormal R_5 1)** **(n=486)**	**Abnormal R_{20} 1)** **(n=456)**	**Abnormal X_5 2)** **(n=434)**
	OR (95% CI)	OR (95% CI)	OR (95% CI)
Numbers of blood biomarkers in upper tertile 4)			
1	0.98 (0.75, 1.26)	0.98 (0.76, 1.28)	1.17 (0.88, 1.54)
2-3	1.04 (0.81, 1.33)	1.02 (0.79, 1.32)	1.29 (0.99, 1.69)
4-6	**1.55 (1.21, 2.00)**	**1.32 (1.01, 1.73)**	**1.97 (1.50, 2.57)**

	**Abnormal A*X* 1)** **(n=455)**	**Abnormal f_{res} 1)** **(n=436)**	**Any abnormal IOS 3)** **(n=1135)**
	OR (95% CI)	OR (95% CI)	OR (95% CI)
1	1.23 (0.94, 1.62)	1.20 (0.91, 1.57)	1.11 (0.93, 1.31)
2-3	1.27 (0.97, 1.65)	**1.33 (1.02, 1.73)**	**1.20 (1.01, 1.42)**
4-6	**1.82 (1.39, 2.39)**	**1.69 (1.28, 2.23)**	**1.56 (1.30, 1.86)**

B.

	**Abnormal R_5 1)** **(n=486)**	**Abnormal R_{20} 1)** **(n=456)**	**Abnormal X_5 2)** **(n=434)**
	OR (95% CI)	OR (95% CI)	OR (95% CI)
Numbers of blood biomarkers in upper tertile 4)			
1	0.94 (0.73, 1.23)	0.97 (0.74, 1.26)	1.16 (0.87, 1.54)
2-3	0.93 (0.72, 1.21)	0.97 (0.74, 1.27)	1.18 (0.89, 1.56)
4-6	1.32 (0.99, 1.76)	1.23 (0.91, 1.66)	**1.75 (1.29, 2.38)**

	**Abnormal A*X* 1)** **(n=455)**	**Abnormal f_{res} 1)** **(n=436)**	**Any abnormal IOS 3)** **(n=1135)**
	OR (95% CI)	OR (95% CI)	OR (95% CI)
1	1.19 (0.90, 1.56)	1.19 (0.90, 1.57)	1.08 (0.91, 1.29)
2-3	1.13 (0.85, 1.49)	1.32 (0.99, 1.74)	1.14 (0.95, 1.36)
4-6	**1.67 (1.23, 2.28)**	**1.71 (1.24, 2.34)**	**1.43 (1.17, 1.75)**

* Adjusted for age, sex, BMI, smoking and study site. Results were statistically significant if the 95% CI does not include 1 (shown in bold)
1) Abnormal R_5, R_{20}, A*X*, f_{res} were defined as indices above the ULN (>95th percentile)
2) Abnormal X_5 was defined as indices below the LLN (<5th percentile)
3) Any abnormal IOS was defined as having either abnormal R_5, R_{20}, A*X*, f_{res} above the ULN (>95th percentile) and/or abnormal X_5 below the LLN (<5th percentile)
4) 1, 2-3 and 4-6 blood biomarkers in the upper tertile were defined as having 1, 2-3 and 4-6 biomarkers (CRP, B-Eos, B-Neu, B-Lym, B-Leu, B-Mono) in the upper tertile, respectively

A*X*: area of reactance, B-Eos: blood eosinophils, B-Neu: blood neutrophils, B-Leu: blood leukocytes, B-Lym: blood lymphocytes, B-Mono: blood monocytes, BMI: body mass index, CI: confidence interval, CRP: C-reactive protein, f_{res}: resonance frequency, Hz: hertz, IOS: impulse oscillometry, LLN: lower limit of normal, n: number, OR: odds ratio, R_5: resistance at 5 Hz, R_{20}: resistance at 20 Hz, ULN: upper limit of normal, X_5: reactance at 5 Hz

Supplementary Table 6. *Adjusted interaction analyses between continuous blood biomarker levels, sex, smoking, body mass index and abnormal impulse oscillometry indices (n=10602).*

	**Abnormal R_5 [1)] (n=722)**	**Abnormal R_{20} [1)] (n=629)**	**Abnormal X_5 [2)] (n=777)**
	OR (95% CI)	OR (95% CI)	OR (95% CI)
Blood biomarkers			
CRP×Female sex	1.01 (0.98, 1.04)	1.01 (0.98, 1.03)	1.01 (0.98, 1.03)
CRP×Smoking	1.02 (0.99, 1.06)	1.03 (0.99, 1.07)	**1.04 (1.00, 1.07)**
CRP×BMI (>30 kg/m^2)	1.00 (0.97, 1.03)	1.00 (0.97, 1.04)	0.99 (0.97, 1.02)
B-Eos×Female sex	1.93 (0.63, 5.91)	2.48 (0.68, 9.07)	1.09 (0.41, 2.87)
B-Eos×Smoking	0.30 (0.07, 1.36)	0.26 (0.04, 1.69)	1.83 (0.50, 6.62)
B-Eos×BMI (>30 kg/m^2)	0.54 (0.14, 2.04)	0.64 (0.14, 2.89)	0.42 (0.14, 1.25)
B-Neu×Female sex	**1.13 (1.01, 1.28)**	1.06 (0.93, 1.20)	**1.13 (1.01 (1.27)**
B-Neu×Smoking	1.02 (0.90, 1.17)	1.14 (0.99, 1.31)	1.04 (0.91, 1.18)
B-Neu×BMI (>30 kg/m^2)	0.92 (0.80, 1.07)	0.91 (0.78, 1.07)	0.94 (0.83, 1.07)
B-Lym×Female sex	1.10 (0.97, 1.26)	1.02 (0.84, 1.25)	1.13 (0.99, 1.28)
B-Lym×Smoking	0.91 (0.80, 1.02)	1.00 (0.84, 1.19)	**0.89 (0.80, 0.99)**
B-Lym×BMI (>30 kg/m^2)	0.82 (0.64, 1.05)	0.79 (0.59, 1.06)	**0.88 (0.78, 0.99)**
B-Leu×Female sex	**1.09 (1.02, 1.16)**	1.05 (0.98, 1.13)	**1.12 (1.05, 1.19)**
B-Leu×Smoking	0.94 (0.88, 1.00)	0.99 (0.92, 1.07)	**0.92 (0.86, 0.98)**
B-Leu×BMI (>30 kg/m^2)	**0.90 (0.82, 0.97)**	0.91 (0.82, 1.01)	**0.89 (0.84, 0.95)**
B-Mono×Female sex	1.72 (0.66, 4.54)	0.90 (0.32, 2.54)	**3.73 (1.47, 9.46)**
B-Mono×Smoking	1.93 (0.65, 5.71)	2.34 (0.70, 7.86)	1.43 (0.49, 4.16)
B-Mono×BMI (>30 kg/m^2)	0.32 (0.10, 1.00)	0.71 (0.22, 2.27)	0.50 (0.18, 1.35)

	**Abnormal AX [1)] (n=790)**	**Abnormal f_{res} [1)] (n=746)**	**Any abnormal IOS [3)] (n=1715)**
	OR (95% CI)	OR (95% CI)	OR (95% CI)
CRP×Female sex	**1.03 (1.00, 1.07)**	**1.04 (1.00, 1.07)**	1.01 (0.99, 1.03)
CRP×Smoking	0.99 (0.96, 1.03)	0.99 (0.96, 1.03)	1.02 (0.99, 1.06)
CRP×BMI (>30 kg/m^2)	0.99 (0.96, 1.02)	0.97 (0.93, 1.01)	1.00 (0.98, 1.03)
B-Eos×Female sex	0.92 (0.34, 2.50)	1.30 (0.47, 3.65)	1.08 (0.51, 2.28)
B-Eos×Smoking	0.86 (0.23, 3.25)	1.10 (0.29, 4.23)	0.82 (0.28, 2.39)
B-Eos×BMI (>30 kg/m^2)	0.62 (0.21, 1.85)	0.58 (0.17, 1.99)	0.68 (0.29, 1.62)
B-Neu×Female sex	**1.24 (1.10, 1.39)**	**1.20 (1.06, 1.35)**	**1.12 (1.03, 1.22)**
B-Neu×Smoking	1.02 (0.90, 1.16)	0.97 (0.86, 1.11)	1.07 (0.97, 1.18)
B-Neu×BMI (>30 kg/m^2)	0.90 (0.79, 1.03)	0.95 (0.82, 1.09)	0.94 (0.85, 1.04)
B-Lym×Female sex	**1.20 (1.05, 1.38)**	**1.16 (1.01, 1.33)**	**1.17 (1.04, 1.31)**
B-Lym×Smoking	**0.87 (0.78, 0.97)**	**0.88 (0.79, 0.98)**	**0.91 (0.83, 0.99)**
B-Lym×BMI (>30 kg/m^2)	**0.84 (0.74, 0.96)**	**0.83 (0.71, 0.98)**	**0.87 (0.79, 0.98)**
B-Leu×Female sex	**1.14 (1.06, 1.22)**	**1.12 (1.05, 1.19)**	**1.11 (1.05, 1.17)**
B-Leu×Smoking	**0.92 (0.87, 0.98)**	**0.92 (0.86, 0.98)**	0.95 (0.90, 1.01)
B-Leu×BMI (>30 kg/m^2)	**0.89 (0.83, 0.95)**	**0.90 (0.84, 0.96)**	**0.91 (0.86, 0.95)**
B-Mono×Female sex	**4.50 (1.76, 11.5)**	**5.58 (2.13, 14.6)**	**2.21 (1.12, 4.36)**
B-Mono×Smoking	0.81 (0.28, 2.39)	0.81 (0.27, 2.43)	1.72 (0.75, 3.93)
B-Mono×BMI (>30 kg/m^2)	0.52 (0.19, 1.42)	0.48 (0.16, 1.48)	0.58 (0.27, 1.23)

* Models adjusted for age, sex, BMI (>30 kg/m^2), smoking and study site. Results were statistically significant if the 95% CI does not include 1 (shown in bold)

1) Abnormal R_5, R_{20}, AX, f_{res} were defined as indices above the ULN (>95th percentile)

2) Abnormal X_5 was defined as indices below the LLN (<5th percentile)

3) Any abnormal IOS was defined as having either abnormal R_5, R_{20}, AX, f_{res} above the ULN (>95th percentile) and/or abnormal X_5 below the LLN (<5th percentile)

AX: area of reactance, B-Eos: blood eosinophils, B-Neu: blood neutrophils, B-Leu: blood leukocytes, B-Lym: blood lymphocytes, B-Mono: blood monocytes, BMI: body mass index, CI: confidence interval, CRP: C-reactive protein, f_{res}: resonance frequency, Hz: hertz, IOS: impulse oscillometry, kg/m^2: kilogram per square metre, LLN: lower limit of normal, n: number, OR: odds ratio, R_5: resistance at 5 Hz, R_{20}: resistance at 20 Hz, ULN: upper limit of normal, X_5: reactance at 5 Hz
